# Artificial Intelligence in Dermatopathology: An Analysis of Its Practical Application

**DOI:** 10.3390/dermatopathology10010014

**Published:** 2023-02-16

**Authors:** Marina Kristy Ibraheim, Rohit Gupta, Jerad M. Gardner, Ashley Elsensohn

**Affiliations:** 1 Department of Dermatology, Loma Linda University, Loma Linda, CA 92354, USA; 2 School of Medicine, Baylor College of Medicine, Houston, TX 77030, USA; 3 Departments of Pathology and Dermatology, Geisinger Medical Center, Danville, PA 17822, USA; 4 Department of Pathology and Human Anatomy, Loma Linda University, Loma Linda, CA 92354, USA

## 1. Introduction

In recent years, researchers have explored potential uses for artificial intelligence (AI) in medical practice. Artificial intelligence (AI) refers to the ability for computer systems to perform tasks that traditionally require human input. Deep learning (DL) entails the creation and layering of multiple artificial neural networks that, when given data to review, allows for self-training and can improve the system’s accuracy.

## 2. Discussion

There is a wealth of data demonstrating a degree of promise for AI in the diagnosis of skin conditions. Xie et al. utilized a set of over 2000 histopathological images to assess the accuracy of two DL architectures in distinguishing melanoma from nevi, finding high overall accuracy, including 92% sensitivity and 94% specificity [1]. Similarly, Cruz-Roa et al. used a set of 1417 histopathological images to create a DL architecture that distinguishes basal cell carcinoma from normal tissue with an accuracy of 91.4% [2].

The sole reliability of AI in dermatopathology practice remains problematic. Convolutional neural networks (CNNs) have been geared to recognize limited types of lesions, such as nevi, seborrheic keratoses, and basal cell carcinoma [3]. Many lesions biopsied in the day-to-day clinical setting fall outside of these categories. Furthermore, dermatopathological diagnoses do not always correlate clinically: lesions that may clinically be thought to be neoplastic may be inflammatory in histopathology. In addition, many histological lesions, such as neoplasms, closely mimic features of one another in clinical practice and require nuanced analyses of slides [4]. Expanding the database of images from which AI learns and develops an archetype may circumvent this to a degree; however, such endeavors would be costly and may require multi-site coordination. Taken together with the fact that current studies demonstrate nearly 10% inaccuracy of diagnosis by AI, it is very likely that dermatopathologists would need to review AI-diagnosed slides. Finally, current studies suggest patients have lower trust in AI making medical diagnoses compared to a physician [5].

In a 2020 international survey of 718 pathologists, the majority of respondents (81.5%) were aware of the emergence of AI in pathology but only a small subset (18.8%) stated that they had good or excellent knowledge about it. Regarding AI diagnostic abilities, 42.6% of respondents felt that there was strong or very strong potential for AI to provide suggested diagnoses for neoplastic skin disease. Although 60.5% of respondents agreed or strongly agreed that AI will revolutionize the field of dermatopathology, only 6.1% believed that human pathologists will be replaced by AI in the foreseeable future [6].

Despite its shortcomings, AI may serve as a practical tool for dermatopathologists in the future. As the technology becomes more refined, it may be used to perform preliminary reads, similar to cardiologists using an electrocardiogram (EKG). This approach may prove helpful in practices with a high volume of slides and paucity of trained dermatopathologists, helping to ensure patients with malignant lesions are diagnosed in a timely manner. For dermatopathologists in the academic setting, AI may function as an intelligent tutoring system that can provide learners with an algorithmic approach to slide review; this may be of benefit to learners when paired with online digital dermatopathology curricula that have boomed in recent years. For researchers, the technology may assist with wide-scale reviews of the electronic medical records through the use of natural language processing [3].

## 3. Conclusions

Widespread utilization of AI in dermatopathology may be far in the future [6,7]. Despite this, AI may begin to serve as a useful tool for triage, education, and research. The success of this technology hinges upon close collaboration among engineers, data scientists, dermatologists, pathologists, and dermatopathologists.

## Data Availability

Not applicable.
